# Bias-adjustment in neuroimaging-based brain age frameworks: A robust scheme

**DOI:** 10.1016/j.nicl.2019.102063

**Published:** 2019-11-04

**Authors:** Iman Beheshti, Scott Nugent, Olivier Potvin, Simon Duchesne

**Affiliations:** a Centre de recherche CERVO, 2601 de la Canardière, Québec, G1J 2G3, Canada.; b Département de radiologie et de médecine nucléaire, Faculté de médecine, Université Laval, 1050, avenue de la Médecine, Québec, G1V 0A6, Canada.

**Keywords:** Brain age, Estimation, Pet, Bias-adjustment, Brain metabolism

## Abstract

The level of prediction error in the brain age estimation frameworks is associated with the authenticity of statistical inference on the basis of regression models. In this paper, we present an efficacious and plain bias-adjustment scheme using chronological age as a covariate through the training set for downgrading the prediction bias in a Brain-age estimation framework. We applied proposed bias-adjustment scheme coupled by a machine learning-based brain age framework on a large set of metabolic brain features acquired from 675 cognitively unimpaired adults through fluorodeoxyglucose positron emission tomography data as the training set to build a robust Brain-age estimation framework. Then, we tested the reliability of proposed bias-adjustment scheme on 75 cognitively unimpaired adults, 561 mild cognitive impairment patients as well as 362 Alzheimer's disease patients as independent test sets. Using the proposed method, we gained a strong R^2^ of 0.81 between the chronological age and brain estimated age, as well as an excellent mean absolute error of 2.66 years on 75 cognitively unimpaired adults as an independent set; whereas an R^2^ of 0.24 and a mean absolute error of 4.71 years was achieved without bias-adjustment. The simulation results demonstrated that the proposed bias-adjustment scheme has a strong capability to diminish prediction error in brain age estimation frameworks for clinical settings.

## Highlights

• We present a robust and simple bias-adjustment scheme for neuroimaging-based brain age frameworks.
• The efficiency of proposed bias-adjustment scheme was assessed in the context of cognitively healthy aging and Alzheimer's disease.
• The proposed bias-adjustment scheme was shown efficient and statistically improved results, making it a necessary part for future brain age frameworks.

## 1 Introduction

“Brain age” estimation through advanced machine learning and brain imaging has become a gripping topic in neuroimaging circles (Cole, Marioni, et al. 2019). The brain age-delta (i.e., Δ: the brain's estimated age minus the individual's chronological age) has been shown as a heritable metric for monitoring cognitively healthy aging, as well as for the early identification of individuals with high-risk of age-associated disorders or mortality (Cole et al., 2018). For example, a brain age delta equal to zero indicates that the individual under study is following a healthy aging trajectory, whereas a higher delta-age would be indicative of an “older-appearing” brain and advanced cognitive aging. However, any accurate judgment about the individual under study forcefully relies on the prediction accuracy of the model.

From a design perspective, most brain age frameworks use a training set of cognitively unimpaired subjects coupled with supervised learning to build this predictive model from brain imaging features as the dependent variable (Franke et al., 2010). Then, for each new sample under study, the estimated brain age is computed by applying the respective brain imaging features to the predicting model. Lately, several brain age estimation studies have reported an age-dependent bias in these predicted results, which contributes to the uncertainty of the interpretation at the clinical level (Le et al., 2018; Cole et al., 2018). Designing and developing a robust and highly accurate brain age prediction framework is therefore needed for any clinical application.

In this study, we propose an adjustment scheme for brain age estimation using fluorodeoxyglucose positron emission tomography (FDG PET) data, as per the technique described by (Goyal et al., 2019)(Sections 2.1–2.3). The adjustment is based on a linear regression model of the brain age bias that includes chronological age as a covariate (Section 2.4). We assessed the proposed bias-adjustment scheme on a large training set of metabolic brain features and compared the results of the proposed adjustment procedure with and without bias-adjustment, as well as the method suggested by Cole (Cole et al., 2018) (Section 3.1). We will show that the proposed bias-adjustment scheme not only removes age-dependency in predicted results, but also can effectively improve the robustness of the brain age prediction results in an independent test set (Section 3.2).

## 2 Material and methods

### 2.1 Participants

We selected adults between the ages of 47 and 94 years from various open and closed source databases to which we had access, namely the Alzheimer's Disease Neuroimaging Initiative (ADNI), Alzheimer's Disease Repository Without Borders (ARWIBO), Banner Alzheimer's Institute (BAI), Centre Hospitalier Universitaire de Sherbrooke (CHUS) and the Open Access Series of Imaging Studies (OASIS). In all studies participants gave informed consent.

In total, 1673 participants were included in the present study including 750 cognitively unimpaired adults, 561 mild cognitive impairment (MCI) patients, and 362 probable Alzheimer's disease (AD) patients. In our simulation, we randomly split the 750 cognitively unimpaired adults into a training set (90%, *N *= 675) and an independent cognitively unimpaired test set (10%, *N* = 75). Furthermore, MCI patients and AD patients were considered as independent test sets.

### 2.2 Image processing

T1-weighted images *-* First, T1-weighted magnetic resonance images (MRI) were obtained for anatomical reference purposes. All T1w MRIs were segmented using *FreeSurfer* 6.0 image analysis suite using the Desikan–Killianny–Tourville (DKT) (Klein and Tourville, 2012) and *FreeSurfer* subcortical atlases with default parameters (http://surfer.nmr.mgh.harvard.edu/). The technical details have been described previously (Fischl and Dale, 2000). In summary, image processing included motion correction, removal of non-brain tissue, automated Talairach transformation, intensity normalization, segmentation of the subcortical white matter and deep grey matter volumetric structures, tessellation of the grey matter and white matter boundary, automated topology correction, and surface deformation to optimally place the grey/white and grey/cerebrospinal fluid boundaries (Reuter et al., 2012).

*FDG PET images -* FDG-PET images that did not have a corresponding T1w MRI acquired within one year of each other were not included in our database. Otherwise, all FDG-PET image pre-processing was performed using the MINC 2.2.00 toolkit. Images were first converted to the MINC2 format, then co-registered to the first frame and timeframe averaged, with the exception of FDG-PET images downloaded from the ADNI database, which were already co-registered to the first frame of the raw image file and timeframe averaged, also known as “post-processed #2” (Jagust et al., 2015). Images were then co-registered to their respective T1w MRI and partial volume corrected (PVC) using region-based voxel-wise correction, an extension of the geometric transfer matrix method. PVC was implemented using PETPVC, which is available on GitHub (https://github.com/UCL/PETPVC). Further details may be found at (Robert et al., 2011). Next, PET images were converted to standard uptake value ratios (SUVR) by the voxel-wise division of the average activity of the paracentral cortex, which had been reported as the optimal region for FDG-PET image normalization in normal aging studies (Jiang et al., 2018). Finally, images were smoothed to a uniform resolution of 8 mm full-width half maximum and the parcellated *FreeSurfer* regions of interest were used to extract estimates of SUVR.

### 2.3 FDG PET based brain age estimation framework

To estimate brain age values, we conducted a prediction model using standard support vector regression algorithm with linear kernel and default settings. For each prediction model, the real age and metabolic brain features were considered as the dependent and independent variables, respectively. We estimated uncorrected brain age using FDG PET data first on the training set (i.e., cognitively unimpaired, *N *= 675) through a k-fold cross validation strategy (*k *= 10). We then calculated the bias-adjusting offset using the entire training set (i.e., cognitively unimpaired, *N *= 675), and then applied the model and the offset to independent test sets (i.e., cognitively unimpaired, *N *= 75; MCI patients, *N* = 561; AD patients, *N* = 362) to estimate brain age in these respective groups.

### 2.4 Proposed bias-adjustment scheme

**Fig. 1:**
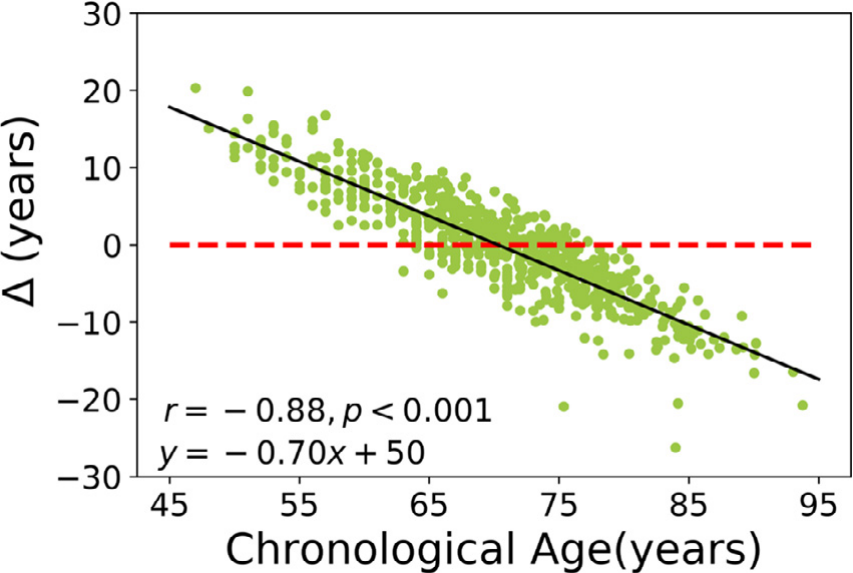
Example of the age-related bias in brain age delta in our training set, where Δ is the estimated brain age minus the real chronological age. The dashed red line shows the reference line (*y *= 0), while the solid black line states the regression line. The result of the training set was generated through a 10-fold-cross validation strategy. (For interpretation of the references to color in this figure legend, the reader is referred to the web version of this article.)

The proposed bias-adjustment scheme relies on the slope (α) and intercept (β) of a linear regression model of brain age delta against chronological age through the training set followed by chronological age as a covariate. To better illustrate the problem at hand, the relationship between brain age delta and chronological age for a training set achieved through a 10-fold cross validation strategy is shown in Fig 1. As can be seen, there is a significant dependence of the brain age delta on chronological age (*r* = −0.88, *p* < 0.001; equation line: *y* = −0.7 x +50). This dependence seems independent of the method being used, as it has been reported by multiple authors (Cole et al., 2019; Boyle et al. 2019) and is likely a result of regression dilution bias (Le et al., 2018). Based on the linear regression line between brain age delta and chronological age, the model either overestimates (i.e., false positive) or underestimates (i.e., false negative) brain age by +16 years to −17 years.

We propose to compute, for each sample under study with a real age of Ω, an offset as follow:

(1) Offset=αΩ+β

where α and β stand respectively for the slope and intercept of a linear regression model of brain age delta against chronological age achieved from a training set. Then, this offset can be subtracted from the individual estimated brain age to achieve a bias-free brain age value for each sample under study.

### 2.5 Alternative method

The technique proposed by Cole et al. is to use the slope and intercept of a linear regression model of estimated brain age versus chronological age obtained from training results(Cole et al., 2018). Indeed, for each sample under study, the bias-free Brain-age value was achieved by subtracting the intercept from predicted brain age and then divided by the slope of a linear regression model of estimated brain age on chronological age obtained from the training set as follow:

(2) $$Predicted_{Bias\;free}=\frac{Predicted_{raw}-\beta }{\alpha }$$

where *Predicted_raw_* stands for predicted brain age. Besides, α and β are the slope and intercept of a linear regression model of estimated brain age as a function of chronological age achieved from a training set, respectively. The bias-adjustment method suggested by Cole (Cole et al., 2018) is considered as a part of a pre-train voxel-based brain age estimator (https://github.com/james-cole/brainageR).

### 2.6 Evaluation and model comparison

The prediction accuracy was assessed on the basis of mean absolute error (MAE), root mean square error (RMSE) and R2 (i.e., coefficient of determination between chronological and estimated brain age). To compare prediction accuracies between procedures, we calculated *p* values based on the MAE confidence intervals (95%) computed from bootstrapping with 1000 random sampling with replacement for the test set.

## 3 Results

### 3.1 Performance measures on training set

We used metabolic-brain features from 675 cognitively unimpaired adults as the training set to build a brain age estimation model, through a 10-fold cross-validation strategy. We then computed the Offset for each subject in the left-out fold in accordance to Eq. (1), and finally report the bias-free brain age value by subtracting the offset from the predicted brain age value.

**Fig. 2:**
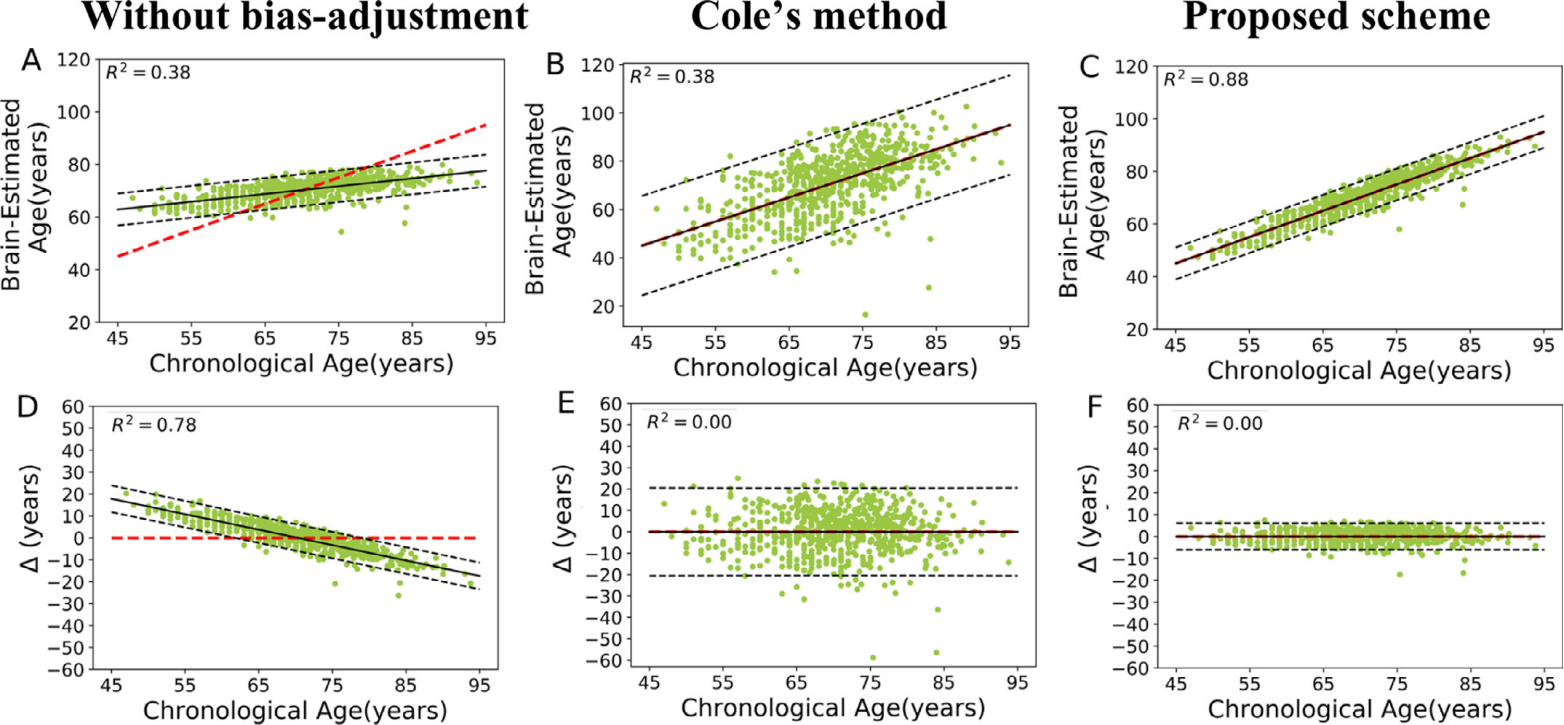
First row (A, B and C): Scatter plot of estimated brain age and chronological age on training set followed by three different procedures. The dashed red line shows the identity line (*y *= *x*), while the dashed black lines state a 95% prediction band on the model prediction. Second row (D, E and F): brain age delta (Δ: estimated brain age minus chronological age) versus chronological age on training set followed by different procedures. The dashed red line shows the reference line (*y *= 0), while the dashed black lines state a 95% prediction band on the model prediction. The results of the training set were generated through a 10-fold-cross validation strategy. (For interpretation of the references to color in this figure legend, the reader is referred to the web version of this article.)

Fig. 2 shows the scatter plot of estimated brain age versus chronological age, as well as the estimated brain age delta versus chronological age for the training set. The prediction accuracies were as follows: without bias-adjustment, an MAE = 5.11 years (RMSE = 6.53 years, R^2^ = 0.38); with Cole's method, an MAE = 8 years (RMSE = 10.39 years, R^2^ = 0.38); and with our proposed method, an MAE = 2.36 years (RMSE = 3.66 years, R^2^ = 0.88). As can be seen from Fig. 2(D), there was a significant age-related variance in the predicted results (i.e., brain age delta vs. chronological age) without bias-adjustment (*r* = −0.88, *p* < 0.001) in the training set. Fig. 2 (E and F) show predicted results (i.e., brain age delta vs. chronological age) after applying bias-adjustment proposed by Cole's method and the proposed scheme. Although both bias-adjustment schemes successfully removed the age-dependency for the predicted results (i.e., *r *= 0, *p *= 1), the proposed scheme significantly diminished the variance further (*F *= 448.88, *p* < 0.001; Levene's test).

### 3.2 Performance measures on test sets

**Fig. 3:**
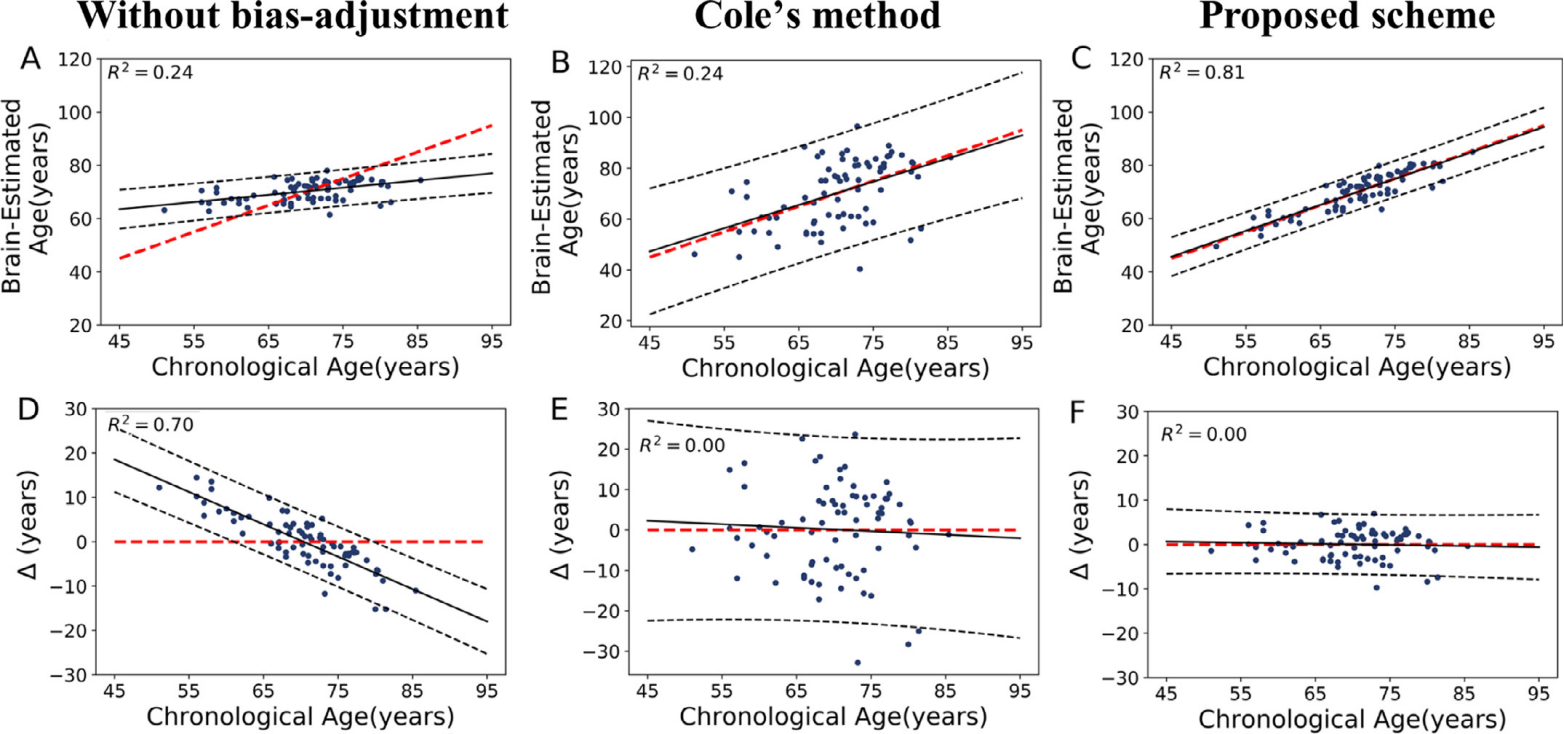
First row (A, B and C): Scatter plot of estimated brain age and chronological age on the independent cognitively unimpaired test set followed by three different procedures. The dashed red line shows the identity line (*y *= *x*), while the dashed black lines state a 95% prediction band on the model prediction. Second row (D, E and F): delta age versus chronological age for the independent cognitively unimpaired set after different procedures. The dashed red line shows the reference line (*y *= 0), while the dashed black lines state a 95% prediction band on the model prediction. (For interpretation of the references to color in this figure legend, the reader is referred to the web version of this article.)

We computed an estimated brain age model and Offset using the complete training set, and then applied this model on the independent test sets. The scatter plot of estimated brain age versus chronological age, as well as brain age delta versus chronological age for the cognitively unimpaired participants (*N *= 75) is shown in Fig. 3. The prediction accuracies followed by different procedures were as follows: without bias-adjustment (MAE = 4.71 years, RMSE = 6.08 years, R^2^ = 0.24), Cole's method (MAE = 9.02 years, RMSE = 11.27 years, R^2^ = 0.24), and the proposed method (MAE = 2.66 years, RMSE = 3.33 years, R^2^ = 0.81). The mean of metabolic brain age delta were: without bias-adjustment 0.20 years [95% confidence intervals (CI) −1.19:1.61], Cole's method 0.13 years [95% CI −2.48:2.74], and our proposed method 0.03 years [95% CI −0.73:0.80]. After applying bias-adjustment, the correlation between brain age delta vs. chronological age was about zero (both methods: *r* = −0.05, *p *= 0.64), in contrast to the significant age-dependency of the uncorrected results (Fig. 3 D; *r* = −0.84, *p* < 0.001). Again, while both methods removed the age bias, our technique reduced variance significantly (*F *= 53.79, *p* < 0.001; Levene's test). Furthermore, the prediction accuracy of the proposed method was significantly superior to without bias-adjustment (*p* < 0.0001), and Cole's method (*p* < 0.0001).

**Fig. 4:**
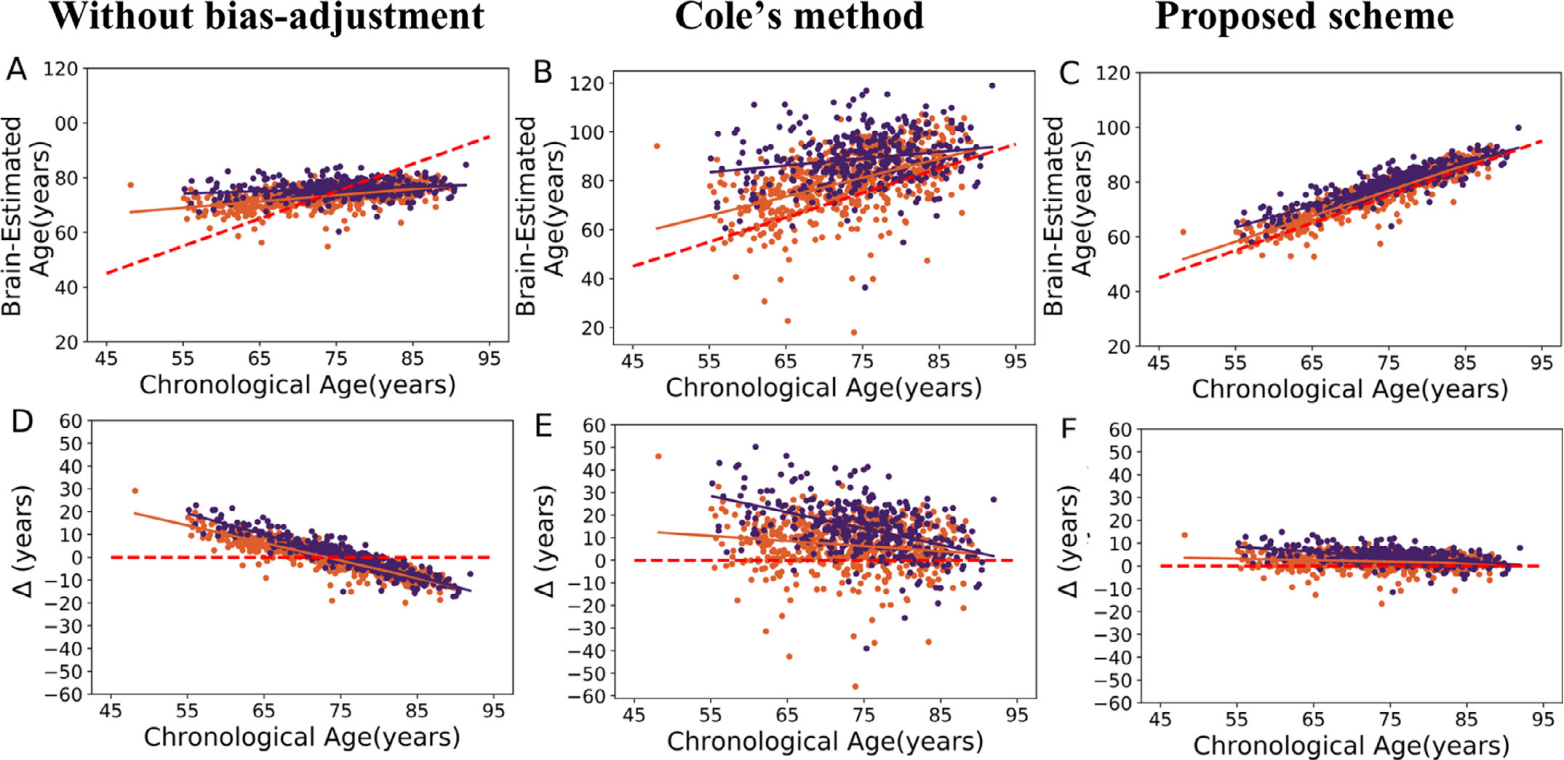
First row (A, B and C): Scatter plot of brain estimated age and chronological age on MCI (orange spot, solid orange regression line) and AD (dark blue spot, solid dark blue regression line) sets followed by different procedures; The dashed red line shows the identity line (*y *= *x*). Second row (D, E and F): delta age versus chronological age on MCI (orange spot, solid orange regression line) and AD (dark blue spot, solid dark blue regression line) followed by different procedures; the dashed red line shows the reference line (*y *= 0). (For interpretation of the references to color in this figure legend, the reader is referred to the web version of this article.)

Fig. 4 illustrates the relationship between the prediction results and chronological age on MCI (*N *= 561) and probable AD (*N *= 362) sets followed by different procedures. The means of metabolic brain age delta values were as follows: without bias-adjustment (MCI = 0.13 years, [95% CI −0.43:0.69]; AD = 0.61 years, [95% CI −0.16:1.40]); Cole's method (MCI= 6.74 years, [95% CI 5.85:7.63]; AD = 13.87 years, [95% CI 12.67:15.08]); and the proposed method (MCI= 2.00 years, [95% CI 1.73:2.25]; AD = 4.09 years, [95% CI 3.74:4.45]). The statistical results showed no significant difference among these independent test groups without bias-adjustment (F(2995) = 0.53, *p *= 0.58; ANOVA, Posthoc analyses using Tukey's HSD), whereas both bias-adjustment methods showed a significant and similar difference in terms of metabolic brain age delta (both methods: (F(2995) = 70, *p* < 0.001; ANOVA, posthoc analyses using Tukey's HSD). Furthermore, we assessed the difference among test groups using bias adjustment technique as proposed in (Le et al., 2018) by including the real age as covariate. According to Le's method, there was a significant difference in terms of metabolic brain age delta among these independent test groups (*F *= 1322, *p* < 0.001; ANOVA, posthoc analyses using Tukey's HSD). Indeed, the MCI and AD subjects showed a conspicuously higher metabolic brain ages compared to the independent cognitively unimpaired set (mean of metabolic brain age delta = 0) regardless of the bias-adjustment method. Both bias-adjustment methods showed the same effect sizes (AD/ cognitively unimpaired: *d *= 1.19; MCI/ cognitively unimpaired: *d *= 0.60). Once again however the variance in the results was significantly smaller for our proposed solution, whereas Cole's technique increased variance from the non-bias adjusted results solution (MCI: *F *= 326.70, *p* < 0.001; AD: *F *= 239.85, *p* < 0.001; Levene's test).

## 4 Discussion

The bias (i.e., age-dependency) is a substantial issue in brain-age frameworks. This bias may be caused by dilution bias (also known as attenuation) of the prediction model, which drives the prediction slope to zero rather than a true slope due to measurement error in the predictors (Young, 2017). This bias therefore has an adverse effect on the outcomes for unseen data. The mathematical details of dilution bias is given in (Young, 2017). To diminish this age-dependency, Cole and colleagues suggested an adjustment procedure as a part of the voxel-wise brain age framework on the basis of the slope and intercept of a linear regression model of estimated brain age versus chronological age (Cole et al., 2018). Cole's method was then successfully applied in a series of voxel-wise brain age estimation studies (Cole et al., 2019). However, when faced with a similar bias, we attempted to apply Cole's method to our PET-FDG data and noticed that it was increasing variance, as well as decreasing accuracy. Therefore, we aimed to design a new method that would not affect nor lower variance, all the while maintaining, if not improving, accuracy. The key idea behind our proposed method is to use the chronological age of each subject under study as a covariate coupled by the slope and intercept of a linear regression model of brain age delta against chronological age driven from the model training set. We used a linear model as a function of real age (Eqn. (1)) to compute the offset for each subject under study as we observed a linear relationship between the bias (i.e., Brain age delta) and chronological age in our training set (Fig. 1). For other modalities that could have a nonlinear relationship with age, our bias-correction method can also be extended using a nonlinear basis functions. Indeed, our proposed bias adjustment only shifts the model slope to a true one rather than a false one due to dilution bias. We expected our proposed bias-adjustment scheme to yield more robust brain-age prediction results than other state-of-the-art bias-adjustment techniques (e.g. (Cole et al., 2018)). The latter removed the age-dependency issue however both training and independent testing results demonstrated that it may not be a robust way to ameliorate the prediction accuracy, as it has a tendency to increase the final variance in the results. Conversely, our proposed bias-adjustment technique achieved a strong R^2^ of 0.81 between chronological age and brain estimated age, an excellent MAE of 2.66 years on independent data, and a statistically reduced variance. In our simulated data, before bias correction, we observed a lower prediction accuracy (R^2^ = 0.38) with a higher age dependency bias on our training set compared to studies using T1-weighted MRI using different techniques (Le et al., 2018; Cole et al., 2017; Beheshti et al. 2019). Thus, the nature of the data predicting age appears to induce more or less age dependency biases. Since FDG-PET data is inducing a prominent bias, the prediction is therefore markedly improved after correcting this bias. The association between brain age delta with clinical features, as a heritable neuroimaging-based metric, has been widely investigated in clinical research (Cole, Underwood, et al. 2017; Cole, Annus, et al. 2017). For example, Cole and colleagues (Cole et al., 2015) explored the association between brain age delta and neuropsychological measures among traumatic brain injury subjects, and reported significant correlations between brain age delta values and memory as well as information processing speed. In another study, the correlation between brain age delta and body mass index as well as intelligence quotient was investigated among adults who suffer from Prader–Willi syndrome (Azor et al. 2019). Of note, the exactness of these analyses relies on the prediction accuracy of the brain age models. While many factors might have a strong impact on prediction results (e.g. scanner properties, imaging modality, pre-processing, training size and population characteristics), the models might also suffer from an age-dependency issue due to regression dilution bias (Le et al., 2018). This bias has been reported in a series of brain age estimation studies (Cole et al., 2019; Boyle et al. 2019; Cole et al., 2018). For instance, Boyle and colleagues (Boyle et al. 2019) reported a significant age-dependency between delta age and chronological age among their training set on the basis of voxel-based features (*r* = −0.44, *p* < 0.001). Using Cole's method, they removed this age-dependency from the prediction results followed by a MAE of 7.28 years on training set (Boyle et al. 2019). Consequently, a robust bias-adjustment technique for achieving bias-free brain age values was needed, which our proposed method seems to accomplish. In this line, statistical testing on brain age delta values among independent data showed that the proposed bias-adjustment scheme has the potential ability of distinguishing clinically different populations (i.e., MCI and AD patients) from cognitively unimpaired participants. It is documented that the brain age frameworks often require large training sets (Franke et al., 2010). Likewise other bias correction methods, a large training set helps to archive more accurate bias adjustment's parameters. Moreover, characteristics such as sex may influence brain-age prediction, thus an unbalanced sample on sex might not be as generalizable as a balanced one. Regarding the difference among these independent test groups, the Le's method and our proposed bias adjustment technique showed a similar significant difference among these independent test groups (*p* < 0.001). However, the Le's technique is appropriate for group comparison only whereas our proposed method is also capable of producing bias free brain age values at the individual level. While our method markedly reduced the variance of the predicted age compared to Cole et al.’s method, both bias-adjustment methods showed the same effect sizes in AD and MCI clinical groups (AD vs cognitively healthy: *d *= 1.19; MCI vs cognitively healthy: *d *= 0.60). Hence, one aiming to compare brain age in MCI or AD and healthy controls can choose either method and should obtain similar results. However, our method is simpler to apply than Cole et al.’s, and more importantly in studies on other clinical groups or with other objectives than group comparison one could benefit from using our approach since it leads to more accurate age predictions.

In this study, we assessed the proposed bias-adjustment scheme on a brain age estimation framework followed by metabolic brain features acquired from PET imaging however, the proposed method can also be readily applied to other brain age frameworks such as voxel-based estimations (Franke et al., 2010), EEG signal-based (Al Zoubi et al. 2018), and patch-based techniques (Beheshti et al. 2019) (see supplementary materials for details). The example Matlab code to compute the brain age delta values through current dataset is available at: https://github.com/medicslab/Bias_Correction.

## 5 Conclusion

In this paper, we presented a unique and simple bias-adjustment scheme as a potential solution to remove age-dependency for predicted brain age results. For each test individual, we computed the respective offset value on the basis of a linear regression model of brain age delta against chronological age driven from a brain FDG PET brain age model using a training set of 675 cognitively unimpaired adults. Following this strategy, the respective offset value was considered to compute the final bias-free brain age value. We assessed the reliability of this proposed bias-adjustment technique on 75 cognitively unimpaired adults, 561 MCI patients and 362 AD patients as independent test sets. We demonstrated that the proposed bias-adjustment scheme has strong potential to be considered as part of a robust brain age framework for a clinical setting.

## Appendix Supplementary materials

Image, application 1
