# Supplementary material for: Bias-adjustment in neuroimaging-based brain age frameworks: A robust scheme
Source: Neuroimage Clin. 2019 Nov 4;24:102063. doi: 10.1016/j.nicl.2019.102063 (PMC6861562; doi:10.1016/j.nicl.2019.102063)
Supplement: Supplementary file 1 [file mmc1.docx]

Supplementary Information

**Bias-adjustment on a T1-weighted structural MRI brain age framework.**

**Material and methods**

To assess our proposed bias-adjustment scheme on a on a T1-weighted structural MRI brain age framework, we used the prediction results from our previous study (i.e., patch-based brain age framework (Beheshti et al. 2019)). In summary, 100 cognitively unimpaired adults were considered as training set and 78 cognitively unimpaired adults as independent test set.

**Results**

**Supplementary Figure 1** shows the scatter plot of estimated brain age versus chronological age, as well as the estimated brain age delta versus chronological age for the training set ( N= 100) for a patch-based brain age framework. The prediction accuracies were as follows: without bias-adjustment, an MAE = 1.60 years (RMSE = 1.94 years, R^2^ = 0.94); with Cole’s method, an MAE 1.62 years (RMSE = 1.98 years, R^2^ = 0.94); and with our proposed method, an MAE = 1.50 years (RMSE = 1.82 years, R^2^ = 0.95).

| **Without bias-adjustment** | **Cole’s method** | **Proposed scheme** |
| --- | --- | --- |
| **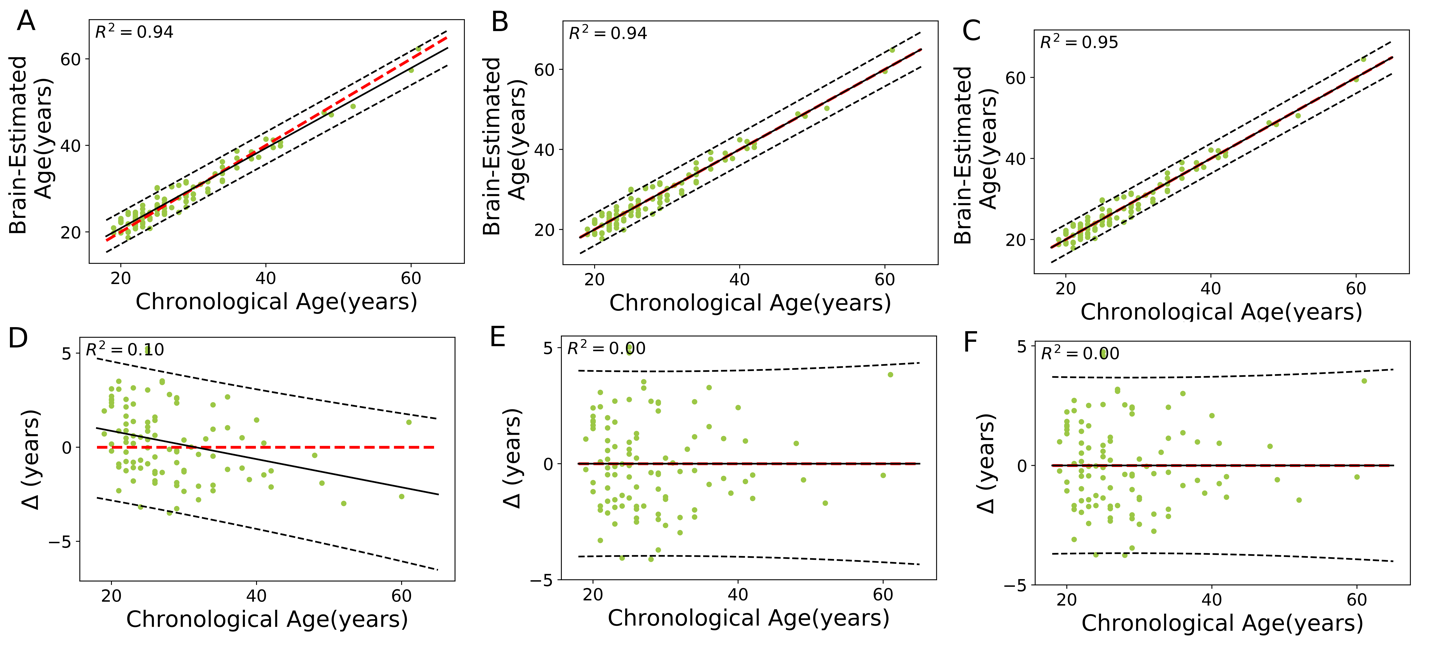** | | |
| **Supplementary Figure 1.** First row (A, B and C): Scatter plot of estimated brain age and chronological age on training set followed by three different procedures for a patch-based brain age framework. The dashed red line shows the identity line (y = x), while the dashed black lines state a 95% prediction band on the model prediction. Second row (D, E and F): brain age delta (Δ : estimated brain age minus chronological age) versus chronological age on training set followed by different procedures. The dashed red line shows the reference line (y = 0), while the dashed black lines state a 95% prediction band on the model prediction. The results of the training set were generated through a leave-one-out strategy. | | |

The scatter plot of estimated brain age versus chronological age, as well as brain age delta versus chronological age for the cognitively unimpaired participants (N = 88) is shown in **Supplementary Figure 2**. The prediction accuracies followed by different procedures were as follows: without bias-adjustment (MAE = 1.66 years, RMSE = 3.00 years, R^2^ = 0.94), Cole’s method (MAE = 1.75 years, RMSE = 3.00 years, R^2^ = 0.94), and the proposed method (MAE = 1.60 years, RMSE = 2.73 years, R^2^ = 0.95).

| **Without bias-adjustment** | **Cole’s method** | **Proposed scheme** |
| --- | --- | --- |
| **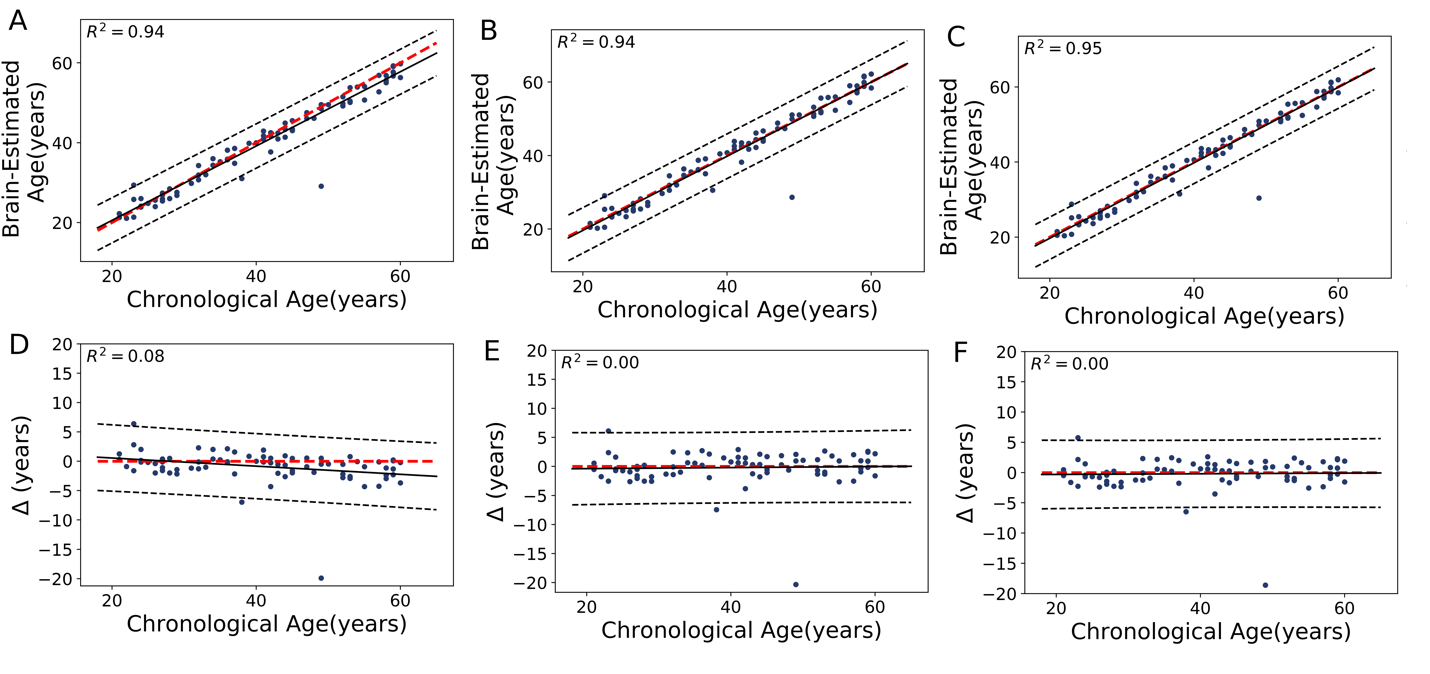** | | |
| **Supplementary Figure 2.** First row (A, B and C): Scatter plot of estimated brain age and chronological age on the independent cognitively unimpaired test set followed by three different procedures for a patch-based brain age framework. The dashed red line shows the identity line (y = x), while the dashed black lines state a 95% prediction band on the model prediction. Second row (D, E and F): delta age versus chronological age for the independent cognitively unimpaired set after different procedures. The dashed red line shows the reference line (y = 0), while the dashed black lines state a 95% prediction band on the model prediction. | | |
